# Trajectories of quality of life and mental health during the Covid-19 lockdown and six months after in Italy. A longitudinal exploration

**DOI:** 10.1007/s10389-023-01913-5

**Published:** 2023-05-03

**Authors:** Guido Veronese, Federica Cavazzoni, Alessandro Pepe

**Affiliations:** 1 Department of Human Sciences “R. Massa”, University of Milano-Bicocca, Piazza dell’Ateneo Nuovo 1, 20126 Milano, Italy; 2 Stellenbosch University, Stellenbosch, South Africa

**Keywords:** Mental health, well-being, quality of life, Fear of Covid-19, anxiety, longitudinal study

## Abstract

**Aim:**

Covid-19 pandemic and its relative containment measures have affected populations' quality of life and psychological well-being worldwide. The fear related to the pandemic and the imposed containment measures has acted as a trigger causing a global increase in negative mental health states. Thus, we aimed to explore the relationship between fear of covid-19 and mental health via QoL (the first and the second lockdown in Italy, 2020).

**Subject and methods:**

Through a quantitative cross-lagged path model research design, the study investigates people’s fear of Covid-19, quality of life, and negative mental states in a population of 444 Italian adults (Mean=40.7; Standard Deviation=16.9; 80% women), in the period between the first and the second waves of the pandemic.

**Results:**

Results show that participants’ Covid-19 fear decreased between waves, contributing to a decrease in negative mental states (stress, anxiety and depression), thus improving the perceived quality of life. Furthermore, quality of life emerged as able to buffer the impact of fear of Covid on people’s psychological distress in short and medium terms, confirming its central role in regulating mental distress.

**Conclusion:**

The study suggests important guidelines for developing interventions to support the populations’ well-being and mental health.

## Introduction

Over two years since Covid-19 began making headlines across the globe, many scholars are concerned about its impact on people's mental health and quality of life. To date, literature has globally documented different factors and aspects related to the pandemic outbreak – and the adopted measures to contain it – that have affected the quality of life and psychological well-being of the populations around the world (Jones et al. 2021; Kola et al. 2021; Liu et al. 2022; O'Connor et al. 2021; Robinson et al. 2022; among others). The containment measures implemented (e.g., social distancing, restriction of movement, closure of nonessential services), combined with the high levels of fear and distress associated with disease contagion, have disrupted people's lives by significantly impacting their mental health and collective well-being (Pakpour and Griffiths 2020; Taylor et al. 2020). Indeed, the World Health Organization has rapidly expressed concerns about the psychosocial consequences of the pandemic, which might be detrimental and long-term (WHO 2021). Up to today, studies have documented a dramatic increase globally, followed by the Covid-19 outbreak, of symptoms of anxiety, stress, insomnia, depression, higher feelings of anger and confusion, panic attacks, emotional exhaustion, and post-traumatic stress reactions (Asmundson and Taylor 2020; Belen 2021; Bhuiyan et al. 2020; Cavazzoni et al. 2022; Meda et al. 2021; Maniaci et al. 2022; Reznik et al. 2020; Satici et al. 2020a, b; Veronese et al. 2021).

## Fear of Covid-19, quality of life and negative mental states

The fear of Covid-19 – also defined as *coronaphobia* (Asmundson and Taylor 2020) – has been outlined among the most influential factors that have significantly impacted people's mental health (Mahmud et al. 2020; Şimşir et al. 2021). The feeling of unpredictability and uncontrollability of the virus [*at the time of data collection, an effective vaccine had not yet been developed*] have been documented as powerful triggers able to exacerbate existing mental frailties, elicit extreme anxiety and stress reactions, foster disproportionate guilt, depressive experiences, and provoke irrational thoughts (Satici et al. 2020a,b; Sun et al. 2020). For instance, several episodes of suicide have been reported as a result of the belief of being infected, contagion later not found in autopsies (Goyal et al. 2020; Mamun and Ullah 2020). Indeed, Covid-19 fear has been documented to increase negative mental states, with a relatively strong impact on people's quality of life (Ahorsu et al. 2020; Veronese et al. 2021).

*Quality of Life* refers to how individuals assess their functioning and satisfaction in multiple domains of their lives. These domains include a sense of emotional control over one's life, social network, satisfaction with one's socioeconomic status, and life fulfilment (Diener et al. 1999). The emergence of the pandemic and the containment measures severely challenged the ability to experience a good quality of life. People's sense of emotional control has been undermined, as has their ability to benefit from their social network (Bruine de Bruin et al. 2020; Cavazzoni et al. 2022). Several studies have highlighted how the lack of social support can harm people's health and mental health (Kafetsios and Sideridis 2006; Lan et al. 2015; Yilmaz et al. 2017). During the quarantine period, social distancing has prevented people from benefiting from their social and family relationships, which is a foundational part of life satisfaction.

Similarly, restraint measures have led to the closure of many activities not considered essential, creating unprecedented impacts on average household income and increasing people's sense of financial instability (Clark et al. 2021; Veronese et al. 2021). In this regard, many studies agreed in highlighting that the most at-risk populations during the pandemic were those with lower income and less education (aspects correlated especially in Western countries), who reported more significant symptoms of anxiety and lowered satisfaction with their lives (Elgar et al. 2020; Solomou and Constantinidou 2020). Furthermore, by referring to demographic factors, the literature has underlined the need for a gender lens when exploring the pandemic impact on people's life and health (Jacques-Aviñó et al. 2020; Ruspini 2020; Sediri et al. 2020; among others). A greater fear of Covid-19, as well as higher symptoms of stress, anxiety, and depression, have been indeed reported by women more than men, with worse outcomes for women's quality of life (Rossi et al. 2020; Wang et al. 2020). Alongside gender, age has also been outlined as a possible risk factor. While the older population is more at risk from a physical standpoint and social isolation (Zysberg and Zisberg 2020), studies have documented that younger age was correlated with greater psychological effects related to the pandemic, with significantly greater anxiety and depression (Wang et al. 2020).

## The study

The present study sought to investigate people's health status and perceptions concerning their quality of life between the first outbreak of the pandemic in Italy (March 2020) and the start of the second wave and lockdown (November 2020). In a recent meta-analysis of longitudinal studies, it was highlighted that the increase in symptoms of stress, anxiety and depression that characterized the first wave of the Covid-19 pandemic did not remain constant in the months that followed, showing significant decreases instead (Robinson et al. 2022). This improvement in mental health in the months after the onset of the pandemic has been evidenced on a large scale (Fancourt et al. 2021; McBride et al. 2021; Gopal et al. 2020; Megalakaki et al. 2021), contextualized by a reduction in Covid-related distress and a decrease in imposed isolation (Fancourt et al. 2021; Daly and Robinson 2021).

In Italy, the first lockdown began in March 2020 with a widespread closure of schools and universities, nonessential activities, movement restrictions, and social distancing. Until May 2020, the population was subjected to several measures deemed necessary for decreasing contagions, such as isolation, restriction of movement, use of protective devices, avoidance of social contacts and encounters, limitation of outdoor sports activities, distance learning and online work. In November 2020, following a surge in infections, the country reintroduced similar but not generalized containment measures. The country was divided into zones (red, yellow, orange, and green) with different restrictions on citizens' freedom of movement. Many schools and universities reintroduced the distance learning mode adopted during the quarantine, and a curfew was introduced. Within the still few longitudinal studies in the Italian territory (Benfante et al. 2022; Salfi et al. 2021), no differences were shown in the population's reported depressive symptoms, compared with a significant reduction in anxiety levels to covid-19 disease. As one of the possible explanations, the authors point out that the decrease in pandemic-related fear allowed for a reduction in anxiety symptoms. Differently, perhaps because the second wave brought back to experience measures of restraint and limitations of freedom – as well as exacerbated economic insecurity – the levels of depression did not exhibit changes.

In this study, we went to investigate the longitudinal chain of relationships between fear of COVID-19, quality of life, and negative mental states in the period between the first and second waves of the pandemic in Italy. Thus, the purpose was to observe whether fear of COVID-19 (FCV-19) decreased between waves (***H1***) and whether this enabled a consequent decrease in negative mental states of anxiety, depression, and stress (***H2***), thereby producing an improvement in perceived quality of life (***H3***). Finally, we tested the function of the quality of life in buffering the effect of fear of the covid-19 on psychological distress (anxiety, depression and stress) both in short and medium terms (***H4***).

## Method

### The sample

We conducted our cross-lagged longitudinal study with 444 Italian participants. At Time 1, the participants' ages varied from 18 to 77 years (M=40.7; SD=16.9); 89 (20%) were males and 355 (80%) were females. In terms of educational level, 2.5 % (n=11) have a lower secondary school diploma, 36.3 % (n=161) have an upper secondary school diploma, 54.5 % (n=242) have a university diploma, and 6.8 % (n=30) have a degree higher than a university diploma. Regarding the general level of health, 22.5% (n=100) of the sample report having some chronic disease (diabetes, hypertension, heart disease, chronic respiratory disease). The criteria for inclusion in the study were (1) having more than 18 years of age, (2) having experienced the pandemic period in Italy, and (3) agreeing to participate in the study.

### Research design and procedure

We used a quantitative cross-lagged path model (CLPM) research design to investigate the longitudinal chain of relationships between fear of COVID-19, quality of life, and negative mental states (for details about CLPM, see Selig and Little 2012). In the social sciences, a CLPM research design is commonly used to assess longitudinal mutual relations among target variables by estimating directional influences over time (Veronese et al. 2020). CLPM models are essential in studying lifespan development because they allow researchers to control for covariates and other potentially confounding variables and evaluate stability effects (concerning previous outcomes scores) (Adachi and Willoughby 2015). They are called "crossed" models because they estimate the path from one variable to another and vice versa, and "lagged" models because they do so over time. To be tested, they must collect a set of quantitative measures from the same participants in at least two waves. As a result, to carry out this design, we administered quantitative self-report measures to our participants during the COVID-19 pandemic emergency using a computer-assisted web interview (CAWI, Couper and Hansen 2002).

The research was approved by the University of Milano-Ethics Bicocca's Board and followed the American Psychological Association's (2010) ethical guidelines, specifically Sections 1 (Ethical Issues), 4 (Privacy and Confidentiality), and 9 (Assessment). The measurements were taken at two points: (1) the time of the first Italian lockdown (March-May 2021) (Time 1), when 500 randomly selected participants completed the study measures throughout an online survey, and (2) and October/December 2021, the second lockdown (Time 2), six months later, when the same subjects were asked to complete the same measures again. Given that 444 participants agreed to participate in both waves, the study's attrition rate was 11.2%.

### Measures (TIME 1)

*Fear of Covid-19 scale*: Fear of COVID-19 Scale (FCV-19). The participants' fear of the coronavirus was measured with the Fear of COVID-19 Scale (FCV-19; Ahorsu et al. 2020). The FCV-7 adopted version (Ahorsu et al. 2020) consists of 7 items (e.g., 'I am most afraid of COVID-19'; 'My heart races or palpitates when I think about getting COVID-19), where ratings are given on a five-point Likert scale, ranging from 1 (strongly disagree) to 5 (strongly agree). In addition, Cronbach's alpha internal consistency coefficients for FCV were calculated at.718.

*World Health Organization Quality of Life* (WHOQOL-BREF; Whoqol Group 1998): The WHOQOL-BREF was a quantitative tool composed of 26 items to evaluate the quality of life (QOL). WHO defines QOL as an individual's perception of their position in life in the context of the culture and value systems in which they live and about their goals, expectations, standards and concerns (Whoqol Group 1998). The questionnaire offered the opportunity to grasp the complexity of the construct by mapping four different domains of the individual perception of QOL: physical health, psychological health, social relationship and environment. Participants are asked to rate their perceptions of various aspects over the last two weeks using a Likert-type response scale (with a five-point granularity). The WHOQOL offered a general quality of life score, with higher values indicating a better condition. For the present study, the Italian version of the questionnaire (available at https://www.who.int/tools/whoqol/whoqol-bref) was administered; the reliability of the cumulate score (as measured by Cronbach's Alpha; Cronbach, 1951) was equal to .793.

*Depression Anxiety Stress Scales Short Version* (DASS-21; Antony et al. 1998): The DASS-21 is a clinical measure initially designed to screen non-clinical samples for the core symptoms of depression and anxiety (Lovibond and Lovibond 1995). The questionnaire includes 21 items that evaluate three types of mental distress symptoms: (a) depression, low self-esteem, and dysphoria; (b) somatic and subjective symptoms of anxiety, as well as fearful reactions; (c) stress, irritability, impatience, tension, and persistent arousal. The questionnaire allows for a cumulative score to represent general levels of negative internal states, with higher scores corresponding to more symptoms. In this study, the Italian version of the questionnaire (Bottesi et al. 2015) was administered; the reliability of the cumulate score (as measured by Cronbach's Alpha; Cronbach, 1951) was equal to .924.

### Measure (time 2)

Six months after Time 1, the participants in the study again completed the fear of COVID-19 scale. WHOQOL-BREF, and DASS-21. The administration procedure was the same for this second wave as for the first. Cronbach's internal reliability for the second wave was: Fear of COVID-19 (α=.793), WHOQOL-BREF (α =.810), and DASS-21 (α =.936).

## Data analysis strategy and quantitative modeling

CLPM was assessed by analyzing the regression and auto-regression coefficients they yielded. The regression coefficients were estimated via structural equation modelling (see Bollen 1989; Mueller and Hancock 2018), including the breakdown of total effects into direct and indirect effects. The Maximum Likelihood method (Gath and Hayes 2006) was adopted to determine the parameters for the structural Equation Modeling (SEM) analysis. The model was evaluated using the following goodness-of-fit indices: χ2 (a not statistically significant chi-square value indicated good fit; Hooper et al. 2008), Tucker–Lewis Index (TLI >0.95; Morin et al. 2013); and comparative fit index (CFI >0.95; Morin et al. 2013). In addition to RMSEA (i.e., the measure is a one-sided test of the null hypothesis that if the RMSEA equals 0.05, the index should not be statistically significant; Kenny et al. 2015) along with the Standardized Root Mean Squared Residual (SRMR). All measures were preliminary checked by computing Mahalanobis' distance (p=.001) to identify and skip multivariate outliers. There were no missing multivariate values in the analysis. The data were also assessed to establish whether the scores were normally distributed. None of the variables under consideration had kurtosis or skewness values that exceeded the recommended limits [2,+2]. (George and Malloy 2010). Amos software was used to test all models (Arbuckle 2003).

**Fig. 1:**
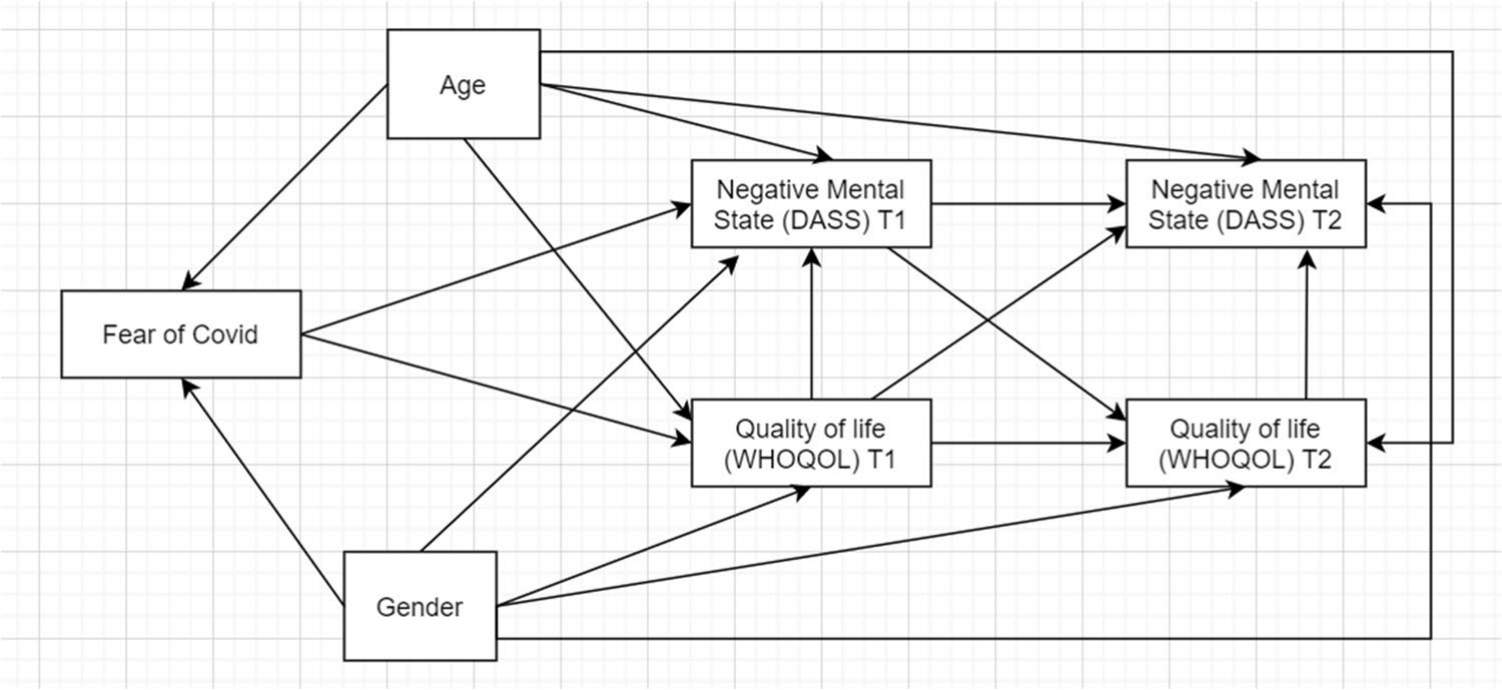
Data analytic strategy. First, a cross-sectional model was specified. Then, the stability model (i.e. with direct paths from measure at Time1 to measures at Time2) was estimated. Finally, the cross-lagged model was evaluated. Cross-lagged effects were represented by direct paths connecting negative mental states at Time1 with quality of life at Time2 and quality of life at Time1 to negative mental states at Time2

A hierarchical testing procedure was used to estimate the causal relationships between fear of COVID-19, quality of life and negative mental states, two measurement points (Meinshausen 2008). We began by analyzing the simplest model and then proceeded to the more complex models, which examine the changes in the goodness of fit values (e.g. NNFI, CFI, RMSEA) at each step in the hierarchy (see Fig. 1).

First, the baseline model (Model A) at Time 1 was evaluated. In line with the research aims, fear of COVID-19 was included in the model as an exogenous variable with direct effects on quality of life and negative mental states as measured at Time 1. Following that, a "stability model" (Model B) was estimated that included direct effects between measures at Time 1 (T1) and Time 2 (T2). Model B was critical in assuming that longitudinal regression paths accounted for causal mechanisms in the observed data (Pearl et al. 2016). The inclusion of autoregressive pathways in CLMPs controls variations in constructs. As a result, an additional model (Model C) with cross-lagged coefficients was tested. Model C included direct effects from the quality of life (as measured at T1) to negative mental states (as measured at T2), as well as negative mental states (at T1) to quality of life (at T2).

Furthermore, we estimated the direct paths from the quality of life and negative mental states at T2 to quality of life and negative mental states at T1. The magnitude of longitudinal effects, according to Adachi and Willoughby (2015), should be evaluated by "putting them in perspective" (p. 126), which means that stability effects should be considered when using bivariate correlations to assess predictive effects. Coherently with the current literature (e.g., MacKinnon et al. 2004), we estimated confidence limits with a set of random samples (k= 500) using both Monte Carlo simulation and bootstrapping methods. We computed the given indirect effects for each of the k samples, as well as the mean value for the chosen pool of samples. The product method was used to calculate indirect effects (MacKinnon et al. 2004). To that end, statistically significant values of at least.05 were considered to indicate a small effect size (Ferguson 2009)

### Controlling variables

In all models, the effects of age and gender were controlled, with direct paths to all exogenous and endogenous variables under study. The inclusion of the demographic characteristics as covariates was based on evidence from the literature and an attempt to compensate for potential sources of confounding relationships. Indeed, life satisfaction and psychological distress have frequently been associated with demographic variables such as age and gender (Bisegger et al. 2005; Lesman-Leegte et al. 2009; Mercier et al. 1998; Myerson et al. 2021; Pepe and Addimando 2013; Rosi et al. 2021; Thomsen et al. 2005; Veronese et al. 2015, 2017; Veronese and Pepe 2020).

## Results

Main statistical descriptives for fear of COVID-19, quality of life and low mental states measures were summarized in Table 1, along with zero-order correlations among all variables in the analysis.

**Table 1:** Summary of zero-order correlations and main descriptive statistics for both T1 and T2 (N=444)

	1	2	3	4	5	6	7
1. Age	-						
2. Gender	-.164**	-					
3. FearOfCovid (T1)	.012	.075	-				
4. Quality of life (WHOQOL; T1)	.037	-.047	-.249**	-			
5. Quality of life (WHOQOL; T2)	.028	-.082	-.225**	.706**	-		
6. Negative Menta States (DASS; T1)	-.119*	-.045	.372**	-.570**	-.470**	-	
7. Negative Menta States (DASS; T2)	-.112*	-.039	.277**	-.455**	-.590**	.693**	-
Mean	40.7	-	3.73	92.3	91.1	13.9	14.1
Standard deviation	16.9	-	2.84	10.8	11.3	9.51	10.1

* *p* < .05, **p < .01

In general terms, zero-order correlations revealed relatively stable patterns of associations. Concerning socio-demographic variables, the gender of participants reports no statistically significant association with all the variables under study, whereas, on the contrary, age is negatively associated with negative internal state scores at both T1 (r=-.12) and T2 (r=-.12). This means that younger people tended to report lower scores of negative internal states than older participants. Looking at the quality of life, the correlations with negative internal states are statistically significant and inverse in both waves. The data indicate in this case that the higher the negative internal states, the lower the quality of life scores (and vice versa). Finally, concerning the stability of scores between T1 and T2, substantial stability can be found with statistically significant correlations ranging between r = .71 (with quality of life ) and r = .69 (with negative internal states).

**Fig. 2:**
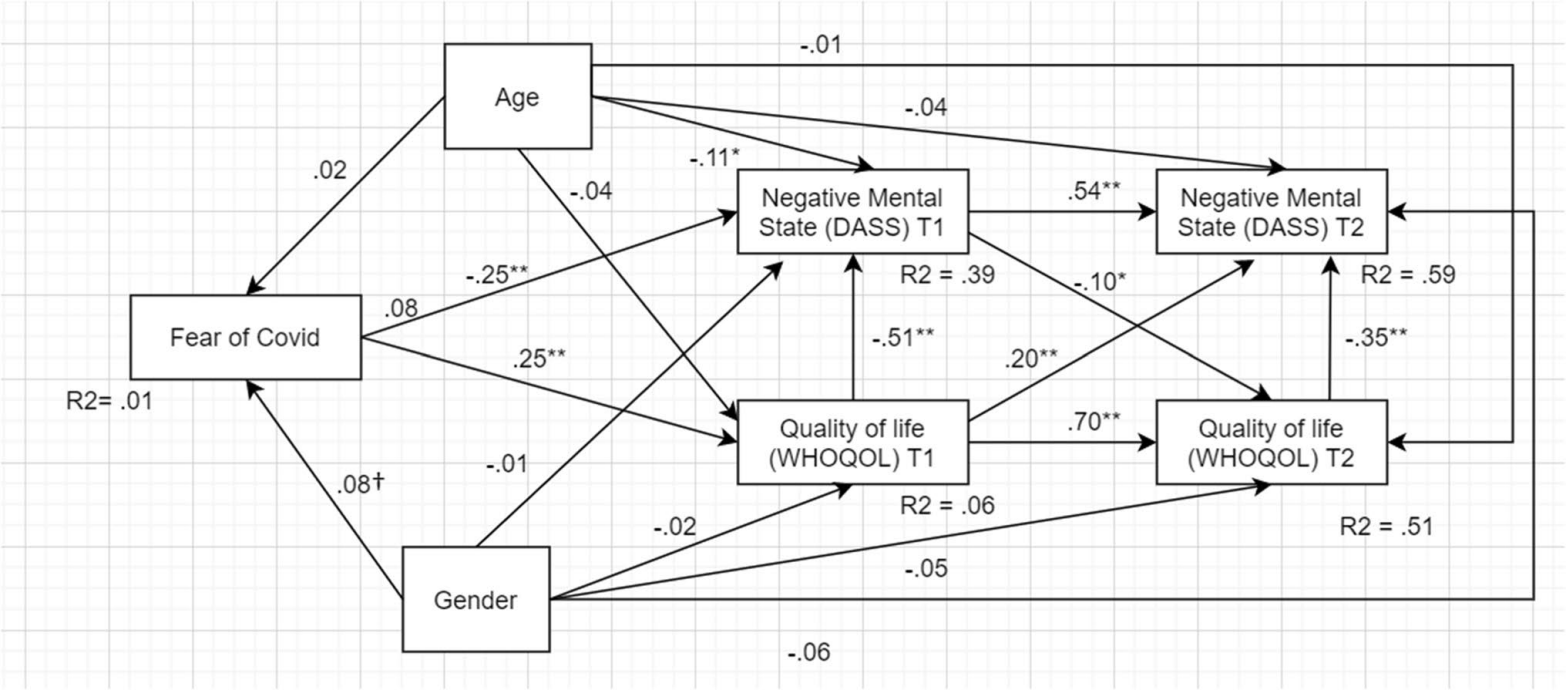
Results of the structural equation model. The cross-lagged path model was estimated on the full sample (N=444). Beta standardized values were reported. * *p* < .05, ** *p* < .01

The next step was to estimate the prospective relationship between fear of Covid-19, quality of life and negative mental states. We first tested the baseline model (Model A), then the model including the stability coefficients between measures gathered at Time 1 and Time 2 (Model B), and finally the cross-lagged model (Model C). All models were controlled for age and gender. The results are reported in Fig. 2.

Evaluation of goodness of fit indexes suggested the acceptance of the cross-sectional model (M1): (χ2(1)=12.09, p=.001; RMSEA=.158, SRMR = .042, NFI=.954, TLI=.956, CFI=.958). Concerning the fit indices, it should be noted that the only value that does not suggest adopting the model is RMSEA. In this regard, however, it should be remembered that the indicator tends to underperform in complex models with few degrees of freedom (Kenny et al. 2015). Next, the stability model (M2) was evaluated. In this case, goodness of fit indexes suggested the full acceptance of M2: (χ2(5)=37.7, *p* < .001; RMSEA=.122, SRMR = .040, NFI=.961, TLI=.966, CFI=.968). Model B's fit with the empirical data suggested that using longitudinal effects in the model resulted in a good representation of the covariances between scores at Time 1 and Time 2 with quality of life and negative mental states. As the last step, we tested the cross-lagged model (Model C), which included the stability coefficients between measures at Time 1 and Time 2 and the cross-lagged paths (R2 values were summarized in Fig. 2). The cross-lagged path model revealed an excellent fit with empirical data: (χ2(3)=12.71, *p* = .003; RMSEA=.085, SRMR = .032, NFI=.987, TLI=.990, CFI=.990) and, all in all, it represented the best model among the three tested.

The main effects emerging from Model C (See Table 2) were summarized in the last part of the results section. Concerning participants' fear of COVID-19, statistically significant total standardized effects were found at T1 between both quality of life at T1(β=-.25, p=.018) and negative mental states (β=.37, p=.008), meaning that the more the fear of the COVID-19, the more participants experienced negative mental states and with less quality of life. Interestingly, the relationship between quality of life and negative mental states is negative (β=.37, p=.009), meaning that quality of life was able to protect from the effect of negative mental states.

**Table 2:** Summary of total, direct and indirect standardized effects of the cross-lagged model (model C)

		Total effect				Direct effect				Indirect effect			
				95% CI				95% CI				95% CI	
From	To	β	p	Lower	Upper	β	p	Lower	Upper	β	p	Lower	Upper
Age	Fear of Covid-19	.025	.696	-0.06	0.103	.025	.696	-0.06	0.103	-	-	-	-
Age	Quality of life T1	.030	.591	-0.054	0.125	.036	.591	-0.043	0.13	-.006	.658	-0.03	0.013
Age	Negative Mental States T1	-.115	.011	-0.199	-0.049	-.106	.011	-0.161	-0.049	-.009	.793	-0.063	0.046
Age	Quality of life T2	.014	.753	-0.065	0.108	-.016	.753	-0.075	0.03	.031	.341	-0.026	0.1
Age	Negative Mental States T2	-.108	.052	-0.182	-0.027	-.04	.052	-0.085	0.025	-.068	.032	-0.143	-0.013
Gender	Fear of Covid-19	.079	.061	0.018	0.153	.079	.061	0.018	0.153	-	-	-	-
Gender	Quality of life T1	-.042	.412	-0.117	0.031	-.023	.412	-0.104	0.048	-.02	.067	-0.04	0.003
Gender	Negative Mental States T1	.026	.667	-0.049	0.106	-.015	.667	-0.079	0.033	.041	.143	-0.005	0.088
Gender	Quality of life T2	-.080	.082	-0.161	-0.004	-.05	.082	-0.109	0.007	-.03	.404	-0.084	0.023
Gender	Negative Mental States T2	.021	.584	-0.039	0.12	-.022	.584	-0.065	0.047	.043	.285	-0.014	0.104
Fear of Covid-19	Quality of life T1	-.248	.018	-0.305	-0.175	-.248	.018	-0.305	-0.175	-	-	-	-
Fear of Covid-19	Negative Mental States T1	.374	.008	0.302	0.441	.249	.008	0.183	0.301	.125	.006	0.094	0.171
Fear of Covid-19	Quality of life T2	-.198	.009	-0.256	-0.149	-	-	-	-	-.198	.009	-0.256	-0.149
Fear of Covid-19	Negative Mental States T2	.261	.006	0.215	0.313	-	-	-	-	.261	.006	0.215	0.313
Quality of Life T1	Negative Mental States T1	-.505	.009	-0.557	-0.449	-.505	.009	-0.557	-0.449	-	-	-	-
Quality of Life T1	Quality of life T2	.698	.013	0.656	0.734	.647	.013	0.599	0.708	.051	.028	0.015	0.084
Quality of Life T1	Negative Mental States T2	-.413	.012	-0.481	-0.344	.205	.012	0.134	0.284	-.618	.006	-0.684	-0.554
Negative Mental States T1	Negative Mental States T2	.638	.035	-0.159	-0.025	.592	.010	-0.159	-0.025	.046	.031	0.012	0.074
Negative Mental States T1	Quality of life T2	-.101	.010	0.573	0.699	-.101	.035	0.519	0.649	-	-	-	-
Quality of Life T2	Negative Mental States T2	-.457	.009	-0.526	-0.394	-.457	.009	-0.526	-0.394	-	-	-	-

β = standardized effect, CI = confidence interval

Similarly, fear of COVID-19 was also found to be less related to the quality of life (β=-.20, p=.009) and negative mental states (β=.26, p=.006) at Time 2. In other words, the direct effect of COVID fear was more significant at T1, while the indirect effect at T2 (i.e. after six months) is attenuated. In both cases, the effects' statistical significance and direction were maintained. In addition, including the cross-lagged paths significantly increased the model's fit. In addition to the path values estimated in the null model, Model C suggested that quality of life and negative mental states were longitudinally interrelated. On the one hand, the results confirmed the stability of the scores across time; in fact, both direct effects on quality of life at Time 1 to quality of life at Time 2 (β=.70, p=.013) and negative mental states at Time 1 and negative mental states at Time 2 (β=.64, *p* = .035) were large, positive, and statistically significant.

In addition, quality of life at Time 1 was statistically related to negative mental states at Time 2 (β=–.41 p=.012), and negative mental states at Time 1 were not statistically related to the quality of life at Time 2 (β=–.10, p=.010). In other words, the more participants displayed a good quality of life, the less they rated negative mental states six months later. On the contrary, the relationship between negative mental states at T1 and quality of life at T2 was less relevant in terms of effect size ( β=–.10, p=.010), highlighting the importance of quality of life in buffering the effect of being afraid of the COVID-19 pandemic on negative mental states both at short terms and medium terms (i.e. after six months) again.

Finally, with regards to participants' demographic, in general, age and gender did not report any statistically significant effects, with the only exception of the direct path from age to negative mental states at T1 (β=–.12, p=.011), whereas at T2 that effects did not report statistical significance.

## Discussion

Our longitudinal study explored the relationship between fear of COVID-19 and mental health via QoL (the first and the second lockdown in Italy 2020) over time. The decrease in fear seemed to augment the participants' mental health (H1, H2) and a better perception of QoL (H3), confirming our hypothesis. Furthermore, quality of life promoted better mental health in Italian adults during the second lockdown.

QoL confirmed a central role in regulating mental distress and fear of COVID-19 in our sample (Ferreira et al. 2021). In Italy, a sudden decline in QoL during the first lockdown created relevant psychological distress among the population, increasing a widespread fear and sense of uncertainty that influenced the population's general mental health (Guida and Carpentieri 2021). The 'stay at home policies', social distancing, and dramatic limitations of economic and social activities had a primary effect of disorientation among the population, increasing levels of anxiety, depression, fatigue, and fear for the future (Epifanio et al. 2021). Moreover, the previous living conditions and QoL determined more risks of developing psychological burdens, making the divide between wealthy and unwealthy people more evident.

This paper showed how QoL might play a crucial role in mental health during the pandemic and the effect of fear on increasing psychological symptoms such as depression, anxiety, and stress (Ornell et al. 2020). Despite the recognized and well-known effects of fear of COVID-19 on psychological distress, we must acknowledge that people with higher QoL might feel more protected and lesser affected by the virus burdens, while people with lower QoL are more exposed to psychological consequences and symptoms (Hansel et al. 2022, Pappa et al. 2022). Accordingly, we must recognize at least two layers of QoL and their influence on people's mental health. First, the pandemic contributed to a general drop in QoL, aggravating the mental distress among the Italian population; secondly, disadvantaged people with an already compromised QoL were more exposed to psychological consequences and the fear of the infection. In sum, the mental health consequences of the pandemic during the one-year acute phase showed an essential role of QoL as a buffering factor protecting the population from psychological symptoms and mitigating the fear of contagion in a historical period in which the health authorities were found to be unprepared to respond to the crisis (Waitzberg et al. 2022).

Some limitations to this study must be discussed and addressed. First, the respondents were mainly women, showing a certain unbalancement in the sample recruitment. However, differences between gender were detected and did not show relevant gaps. Online recruitment should have limited access to the strip of the population technologically disadvantaged and, consequently, with lower QoL. Qualitative future research might help see differences and potentially aggravating factors in people belonging to the most socioeconomic disadvantaged part of the population.

## Conclusions

The Italian authority policies on COVID-19 contrast have been focused on two pillars. Response to the health emergency first, and mitigation of the psychological consequences. The two levels were considered dramatically imbricated (Pompili et al. 2021). However, authorities seemed to have underestimated people's QoL in such a dyad. The severe restrictions have affected the population's QoL, aggravating their mental conditions sensibly. Following the holistic WHO's definition of health and mental health, the lesson learned from our study depicts the Italian population's mental health and well-being as strictly related to their quality of life (Andrei et al. 2022). Psychological services, emergency psychological hotlines, and psychiatry-oriented interventions to contrast the psychological consequences of the pandemic might be insufficient if the national authorities will not orient their efforts in promoting people's QoL during and after the COVID-19 crisis (Dosi and Soete 2022). The syndemic nature of the COVID-19 outbreak requires at national and international levels more attention to disparities, promotion of opportunities and life quality improvement matched with health technologies and initiatives oriented at promoting public mental health. Reducing the pandemic to sole health and a psychiatric issue might result in a limitation exposing the population to more significant risks for their mental health.

## Data Availability

The datasets generated and analyzed during the current study are available from the corresponding author on reasonable request.

## References

[CR1] Adachi P, Willoughby T (2015). Interpreting effect sizes when controlling for stability effects in longitudinal autoregressive models: Implications for psychological science. Eu J Develop Psych.

[CR2] Ahorsu DK, Lin CY, Imani V, Saffari M, Griffiths MD, Pakpour AH (2020) The fear of COVID-19 scale: Development and initial validation. Int J Men Health and Add 1–9. 10.1007/s11469-020-00270-8 10.1007/s11469-020-00270-8 PMC7100496 32226353

[CR3] American Psychological Association (2010). Amendments to the 2002. Ethical principles for psychologists and code of conduct. Am Psycho.

[CR4] Andrei F, Mancini G, Agostini F, Epifanio MS, Piombo MA, Riolo M et al (2022) Quality of life and job loss during the COVID-19 pandemic: mediation by hopelessness and moderation by trait emotional intelligence. Int J Environ Res Public Health 19:2756. 10.3390/ijerph19052756 10.3390/ijerph19052756 PMC8910407 35270449

[CR5] Antony MM, Bieling PJ, Cox BJ, Enns MW, Swinson RP (1998) Psychometric properties of the 42-item and 21-item versions of the Depression Anxiety Stress Scales in clinical groups and a community sample. Psychol Assess 10(2):176–181

[CR6] Arbuckle J (2003) Amos 5.0 update to the Amos user's guide. Marketing Department, SPSS Incorporated

[CR7] Asmundson GJ, Taylor S (2020). Coronaphobia: Fear and the 2019-nCoV outbreak. J anx dis.

[CR8] Belen H (2021) Fear of COVID-19 and mental health: The role of mindfulness in during times of crisis. Int J Ment Health Add 1–12. 10.1007/s11469-020-00470-2 10.1007/s11469-020-00470-2 PMC8075278 33935608

[CR9] Benfante A, Tesio V, Di Tella M, Romeo A, Castelli L (2022). From the first to the second wave of COVID-19: anxiety, de-pressive, and post-traumatic stress symptoms in the Italian population. Int J of En Res Pub Health.

[CR10] Bhuiyan AI, Sakib N, Pakpour AH, Griffiths MD, Mamun MA (2020). COVID-19- related suicides in Bangladesh due to lockdown and economic factors: Case study evidence from media reports. Int J M Health Add.

[CR11] Bisegger C, Cloetta B, Von Bisegger U, Abel T, Ravens-Sieberer U (2005). Health-related quality of life: gender differences in childhood and adolescence. Sozial-und Präventivmedizin.

[CR12] Bollen KA (1989). Structural equations with latent variables.

[CR13] Bottesi G, Ghisi M, Altoè G, Conforti E, Melli G, Sica C (2015). The Italian version of the Depression Anxiety Stress Scales-21: Factor structure and psychometric properties on community and clinical samples. Comp psych.

[CR14] Bruine de Bruin W, Parker AM, Strough J (2020). Age differences in reported social networks and well-being. Psych and Ag.

[CR15] Cavazzoni F, Pancake R, Veronese G (2022) Impact of COVID-19 Pandemic on Mental Health and Quality of Life. An Exploratory Study During the First Outbreak in Italy. Psychol Rep. 10.1177/00332941211066259 10.1177/00332941211066259 PMC8914301 35271789

[CR16] Clark, A, Ambrosio C, Lepinteur A (2021) The fall in income inequality during COVID-19 in five European countries. Archives-ouvertes.fr. halshs-03185534 10.1007/s10888-021-09499-2 PMC8349307 34393688

[CR17] Couper MP, Hansen SE (2002). Computer-assisted interviewing.

[CR18] Cronbach LJ (1951). Coefficient alpha and the internal structure of tests. Psychom.

[CR19] Daly M, Robinson E (2021) Longitudinal changes in psychological distress in the UK from 2019 to September 2020 during the COVID-19 pandemic: Evidence from a large nationally representative study. Psychiatry res. 10.1016/j.psychres.2021.113920 10.1016/j.psychres.2021.113920 PMC9755113 33882397

[CR20] Diener E, Suh EM, Lucas RE, Smith HL (1999). Subjective well-being: Three decades of progress. Psych Bull.

[CR21] Dosi G, Soete L (2022) On the syndemic nature of crises: A Freeman perspective. Res Policy 51. 10.1016/j.respol.2021.104393 10.1016/j.respol.2021.104393 PMC8511438 34658456

[CR22] Elgar FJ, Stefaniak A, Wohl MJ (2020). The trouble with trust: Time-series analysis of social capital, income inequality, and COVID-19 deaths in 84 countries. Soc Sci Med.

[CR23] Epifanio MS, Andrei F, Mancini G, Agostini F, Piombo MA, Spicuzza V (2021). The impact of COVID-19 pandemic and lockdown measures on quality of life among Italian general population. J Clin Med.

[CR24] Fancourt D, Steptoe A, Bu F (2021). Trajectories of anxiety and depressive symptoms during enforced isolation due to COVID-19 in England: a longitudinal observational study. Lancet Psychiatry.

[CR25] Ferguson CJ (2009). Is psychological research really as good as medical research? Effect size comparisons between psychology and medicine. Review Gen Psych.

[CR26] Ferreira LN, Pereira LN, da Fé BM, Ilchuk K (2021). Quality of life under the COVID-19 quarantine. Qual Life Res.

[CR27] Gath EG, Hayes K (2006). Bounds for the largest Mahalanobis distance. Linear Algebra Appl.

[CR28] George D, Malloy M (2010) SPSS for Windows step by step: A simple guide and reference, 17.0 update (10a ed.). Pearson, Boston, MA

[CR29] Gopal A, Sharma AJ, Subramanyam MA (2020). Dynamics of psychological responses to COVID-19 in India: A longitudinal study. PLoS ONE.

[CR30] Goyal P, Choi JJ, Pinheiro LC, Schenck EJ, Chen R, Jabri A (2020). Clinical characteristics of Covid-19 in New York city. N Eng J Med.

[CR31] Guida C, Carpentieri G (2021) Quality of life in the urban environment and primary health services for the elderly during the Covid-19 pandemic: An application to the city of Milan (Italy). Cities, 110, 10.1016/j.cities.2020.103038. 10.1016/j.cities.2020.103038 PMC7691131 33262550

[CR32] Hansel TC, Saltzman LY, Melton PA, Clark TL, Bordnick PS (2022). COVID-19 behavioral health and quality of life. Scientific Reports.

[CR33] Hooper D, Coughlan J, Mullen M (2008, September) Evaluating model fit: a synthesis of the structural equation modelling literature. In 7th European Conference on research methodology for business and management studies (pp. 195-200).

[CR34] Jacques-Aviñó C, López-Jiménez T, Medina-Perucha L, De Bont J, Gonçalves AQ, Duarte-Salles T, Berenguera A (2020). Gender-based approach on the social impact and mental health in Spain during COVID-19 lockdown: a cross-sectional study. BMJ open.

[CR35] Jones EA, Mitra AK, Bhuiyan AR (2021). Impact of COVID-19 on mental health in adolescents: A systematic review. In J Envir Res Public Health.

[CR36] Kafetsios K, Sideridis GD (2006). Attachment, social support and well-being in young and older adults. J Health Psych.

[CR37] Kenny DA, Kaniskan B, McCoach DB (2015). The performance of RMSEA in models with small degrees of freedom. Soc Met Res.

[CR38] Kola L, Kohrt BA, Hanlon C, Naslund JA, Sikander S, Balaji M (2021). COVID-19 mental health impact and responses in low-income and middle-income countries: reimagining global mental health. Lancet Psychiatry.

[CR39] Lan G, Yuan Z, Cook A, Xu Q, Jiang H, Zheng H, Wang L, Yuan L, Xie X, Lu Y (2015). The relationships among social support and quality of life in persons living with HIV/AIDS in Jiangxi and Zhejiang provinces China. AIDS Care.

[CR40] Lesman-Leegte I, Jaarsma T, Coyne JC, Hillege HL, Van Veldhuisen DJ, Sanderman R (2009). Quality of life and depressive symptoms in the elderly: a comparison between patients with heart failure and age-and gender-matched community controls. J Card Fail.

[CR41] Liu Q, Liu Z, Lin S, Zhao P (2022) Perceived accessibility and mental health consequences of COVID-19 containment policies. J Transp Health 101354. 10.1016/j.jth.2022.101354 10.1016/j.jth.2022.101354 PMC8882410 35251936

[CR42] Lovibond PF, Lovibond SH (1995). The structure of negative emotional states: Comparison of the Depression Anxiety Stress Scales (DASS) with the Beck Depression and Anxiety Inventories. Behav res and ther.

[CR43] MacKinnon DP, Lockwood CM, Williams J (2004). Confidence limits for the indirect effect: Distribution of the product and resampling methods. Multivariate Behav Res.

[CR44] Mahmud MS, Talukder MU, Rahman SM (2020). Does 'Fear of COVID-19' trigger future career anxiety? An empirical investigation considering depression from COVID-19 as a mediator. Int J Social Psychiat.

[CR45] Mamun MA, Ullah I (2020). COVID-19 suicides in Pakistan, dying off not COVID-19 fear but poverty? The forthcoming economic challenges for a developing country. Brain Behav Immun.

[CR46] Maniaci G, La Cascia C, Giammanco A, Maria C, Ferraro L, Tripoli G et al (2022) Latent burnout profiles in a sample of frontline healthcare professionals after the peak of the Italian COVID-19 pandemic. Med J Clin Psychol 10. 10.1177/0020764020935488

[CR47] McBride O, Murphy J, Shevlin M, Gibson-Miller J, Hartman TK, Hyland P (2021). Monitoring the psychological, social, and economic impact of the COVID-19 pandemic in the population: Context, design and conduct of the longitudinal COVID-19 psychological research consortium (C19PRC) study. Int J Methods Psychiatr Res.

[CR48] Meda N, Pardini S, Slongo I, Bodini L, Zordan MA, Rigobello P (2021). Students' mental health problems before, during, and after COVID-19 lockdown in Italy. J Psychiatr Res.

[CR49] Megalakaki O, Kokou-Kpolou CK, Vaudé PS, Iorfa SK, Cénat JM, Derivois D (2021) Does peritraumatic distress predict PTSD, depression and anxiety symptoms during and after COVID-19 lockdown in France? A prospective longitudinal study. J Psychiatr Res 137. 10.1016/j.jpsychires.2021.02.035 10.1016/j.jpsychires.2021.02.035 PMC7885671 33662655

[CR50] Meinshausen N (2008). Hierarchical testing of variable importance. Biometrika.

[CR51] Mercier C, Peladeau N, Tempier R (1998). Age, gender and quality of life. Commun Mental Health J.

[CR52] Morin AJS, Marsh HW, Nagengast B (2013) Exploratory structural equation modeling: an introduction. In: Hancock GR, Mueller RO (eds) Structural equation modeling: a second course, 2nd edn. IAP, Greenwich, CT, pp 395–436

[CR53] Mueller RO, Hancock GR, Hancock GR, Stapleton LM, Mueller RO (2018). Structural equation modeling. The reviewer's guide to quantitative methods in the social sciences.

[CR54] Myerson J, Strube MJ, Green L, Hale S (2021). Individual differences in COVID-19 mitigation behaviors: The roles of age, gender, psychological state, and financial status. Plos One.

[CR55] O'Connor RC, Wetherall K, Cleare S, McClelland H, Melson AJ, Niedzwiedz CL (2021). Mental health and well-being during the COVID-19 pandemic: longitudinal analyses of adults in the UK COVID-19 Mental Health & Wellbeing study. Br J Psychiat.

[CR56] Ornell F, Schuch JB, Sordi AO, Kessler FHP (2020). Pandemic fear and COVID-19: mental health burden and strategies. Braz J Psychiat.

[CR57] Pakpour AH, Griffiths MD (2020). The fear of COVID-19 and its role in preventive behaviors. J Concurr Disor.

[CR58] Pappa S, Barmparessou Z, Athanasiou N, Sakka E, Eleftheriou K, Patrinos S (2022). Depression, Insomnia and Post-Traumatic Stress Disorder in COVID-19 Survivors: Role of Gender and Impact on Quality of Life. J Person Med.

[CR59] Pearl J, Glymour M, Jewell NP (2016) Causal inference in statistics: a primer. Wiley

[CR60] Pepe A, Addimando L (2013). Comparison of occupational stress in response to challenging behaviours between general and special education primary teachers in Northern Italy. Int J Special Edu.

[CR61] Pompili M, Innamorati M, Sampogna G, Albert U, Carmassi C, Carrà, G, Cirulli F, Erbuto D, Luciano M, Nanni MG, Sani G, Tortorella, A, Viganò C, Volpe U, Fiorillo A (2021) The impact of Covid-19 on unemployment across Italy: consequences for those affected by psychiatric conditions. J Affect Disord 296:59–66. 10.1016/j.jad.2021.09.035 10.1016/j.jad.2021.09.035 PMC8445771 34592657

[CR62] Reznik A, Gritsenko V, Konstantinov V, Khamenka N, Isralowitz R (2020). COVID-19 fear in Eastern Europe: Validation of the fear of COVID-19 scale. Int J Mental Health Addic.

[CR63] Robinson E, Sutin AR, Daly M, Jones A (2022). A systematic review and meta-analysis of longitudinal cohort studies comparing mental health before versus during the COVID-19 pandemic in 2020. J Affec Disor.

[CR64] Rosi A, Van Vugt FT, Lecce S, Ceccato I, Vallarino M, Rapisarda F (2021). Risk perception in a real-world situation (COVID-19): how it changes from 18 to 87 years old. Fron Psychol.

[CR65] Rossi R, Socci V, Talevi D, Mensi S, Niolu C, Pacitti F, Di Lorenzo G (2020). COVID-19 pandemic and lockdown measures impact on mental health among the general population in Italy. Fron Psychiat.

[CR66] Ruspini E (2020) L'emergenza Covid-19 attraverso la lente del genere. Sicurezza e scienze sociali 42-58. 10.3280/SISS2020-002005

[CR67] Salfi F, D'Atri TD, Ferrara M (2021). Sleeping under the waves: A longitudinal study across the contagion peaks of the COVID-19 pandemic in Italy. J Sleep Res.

[CR68] Satici B, Saricali M, Satici SA, Griffiths MD (2020a) Intolerance of uncertainty and mental well-being: serial mediation by rumination and fear of COVID-19. Int J Mental Health Addict 1–12. 10.1007/s11469-020-00305-0 10.1007/s11469-020-00305-0 PMC7228430 32427165

[CR69] Satici B, Gocet-Tekin E, Deniz ME, Satici SA (2020b) Adaptation of the Fear of COVID-19 Scale: Its association with psychological distress and life satisfaction in Turkey. Int J Mental Health Addict 1–9. 10.1007/s11469-020-00294-0 10.1007/s11469-020-00294-0 PMC7207987 32395095

[CR70] Sediri S, Zgueb Y, Ouanes S, Ouali U, Bourgou S, Jomli R, Nacef F (2020). Women's mental health: acute impact of COVID-19 pandemic on domestic violence. Arch Women Ment Health.

[CR71] Selig JP, Little TD, Laursen BT, Little D, Card NA (2012). Autoregressive and cross-lagged panel analysis for longitudinal data. Handbook of developmental research methods.

[CR72] Şimşir Z, Koç H, Seki T, Griffiths MD (2021) The relationship between fear of COVID 19 and mental health problems: a meta-analysis. Death Stud 1-9. 10.1080/07481187.2021.1889097 10.1080/07481187.2021.1889097 33641626

[CR73] Solomou I, Constantinidou F (2020). Prevalence and predictors of anxiety and depression symptoms during the COVID-19 pandemic and compliance with precautionary measures: Age and sex matter. Int J Env Res Public Health.

[CR74] Sun P, Lu X, Xu C, Sun W, Pan B (2020). Understanding of COVID-19 based on current evidence. J Med Virol.

[CR75] Taylor S, Landry CA, Paluszek MM, Fergus TA, McKay D, Asmundson GJ (2020). COVID stress syndrome: Concept, structure, and correlates. Dep Anx.

[CR76] Thomsen DK, Mehlsen MY, Viidik A, Sommerlund B, Zachariae R (2005). Age and gender differences in negative affect—Is there a role for emotion regulation?. Person Individ Diff.

[CR77] Veronese G, Pepe A (2020). Cross-cultural adaptation, psychometric proprieties and factor structure of the Multidimensional Student Life Satisfaction Scale (MSLSS): A study with Palestinian children living in refugee camps. Current Psychol.

[CR78] Veronese G, Pepe A, Jaradah A, Murannak F, Hamdouna H (2015) Quality of life and determinants of parents' school satisfaction in war contexts: A mixed-method exploratory study in Palestine. Sage Open 5(4). 10.1177/2158244015608422

[CR79] Veronese G, Pepe A, Dagdouke J, Addimando L, Yagi S (2017). Measuring well-being in Israel and Palestine: the subjective well-being assessment scale. Psychol Rep.

[CR80] Veronese G, Pepe A, Obaid H, Cavazzoni F, Perez J (2020). Agency and life satisfaction in Bedouin children exposed to conditions of chronic stress and military violence: A two-wave longitudinal study in Palestine. Clin Child Psychol Psychiatry.

[CR81] Veronese G, Cavazzoni F, Fiore F, Pancake R (2021). Fear of COVID-19 mediates the relation between mental distress and at-risk health behaviours in Italian adults. Med J Clin Psycho.

[CR82] Waitzberg R, Gerkens S, Dimova A, Bryndová L, Vrangbæk K, Jervelund SS, Birk HO, Rajan S, Habicht T, Tynkkynen LK, Keskimäki I, Or Z, Gandré C, Winkelmann J, Ricciardi W, de Belvis AG, Poscia A, Morsella A, Slapšinskaitė A, Miščikienė L, …, Quentin W (2022) Balancing financial incentives during COVID-19: a comparison of provider payment adjustments across 20 countries. Health Policy (Amsterdam, Netherlands) 126(5):398–407. 10.1016/j.healthpol.2021.09.015 10.1016/j.healthpol.2021.09.015 PMC8492384 34711443

[CR83] Wang Y, Di Y, Ye J, Wei W (2020). Study on the public psychological states and its related factors during the outbreak of coronavirus disease 2019 (COVID-19) in some regions of China. Psychol, Health Med.

[CR84] Whoqol Group (1998). Development of the World Health Organization WHOQOL-BREF quality of life assessment. Psychol Med.

[CR85] World Health Organization (WHO) (2021) WHO coronavirus (COVID-19) dashboard. https://COVID19.who.int/

[CR86] Yilmaz MS, Piyal B, Akdur R (2017). Social support and quality of life in a group of cancer patients (Ankara, Turkey). Turkish J Med Scie.

[CR87] Zysberg L, Zisberg A (2020) Days of worry: Emotional intelligence and social support mediate worry in the COVID-19 pandemic. J Health Psychol 1–10. 10.1177/1359105320949935 10.1177/1359105320949935 32811195

